# The use of integrative therapy based on Yoga and Ayurveda in the treatment of a high-risk case of COVID-19/SARS-CoV-2 with multiple comorbidities: a case report

**DOI:** 10.1186/s13256-020-02624-1

**Published:** 2021-02-24

**Authors:** Alka Mishra, Sumitra A. Bentur, Sonika Thakral, Rahul Garg, Bhanu Duggal

**Affiliations:** 1 Department of Ayurveda and Holistic Health, Dev Sanskriti Vishwavidyalaya, Haridwar, India; 2 Ayurveda physician and yoga expert, Noida, India; 3 Shaheed Sukhdev College of Business Studies, University of Delhi, Delhi, India; 4 National Resource Center for Value Education in Engineering, Indian Institute of Technology, Delhi, India; 5 Department of Cardiology, All India Institute of Medical Sciences, Rishikesh, India; 6 Amar Nath and Shashi Khosla School of Information Technology, Indian Institute of Technology, Delhi, India; 7 Department of Computer Science and Engineering, Indian Institute of Technology, Delhi, India

**Keywords:** COVID-19, Ayurveda, Yoga, Integrative therapy, Case report

## Abstract

**Background:**

We report a high-risk case of a coronavirus disease 19 (COVID-19)-positive patient with comorbidities including diabetes mellitus (DM), hypertension (HTN), hypothyroidism and chronic kidney disease (CKD), treated successfully using an integrative therapy plan based on Ayurveda and Yoga, along with government-mandated compulsory modern western medicine (MWM) treatment. Recently, some evidence has been emerging on the use of Ayurveda for treatment of COVID-19. The classical texts of Ayurvedic medicine such as *Charaka Samhita* and *Sushruta Samhita* contain descriptions of pandemics of similar proportions and describe them as *Janapadoddhvansa*, meaning the destruction of communities, along with their causes and treatment.

**Case presentation:**

The case reported herein is a 55-year-old man from Delhi, India, with confirmed (tested) COVID-19, who first took MWM for 7 days before seeking integrative therapy. The patient has comorbidities including DM, HTN, hypothyroidism and CKD and had developed symptoms including fever (which was resolved by the time integrative therapy was started), sore throat, dry cough, body aches, weakness, bad taste and smell, and heaviness in the abdomen. Based on the patient’s symptoms and comorbidities, a treatment plan including Ayurvedic medicines, Yoga protocol, dietary recommendations and lifestyle modifications was prescribed by a registered Ayurveda doctor and a Yoga consultant. The patient started experiencing improvement in all the symptoms within 2 days after starting the treatment; he reported approximately 75% relief from the symptoms after 5 days, and almost complete relief within 9 days. Also, the blood sugar levels (both fasting blood sugar [FBS] and postprandial blood sugar [PPBS]) exhibited significant improvement after 5 days, and decreased to within the normal range within 12 days. Besides relief in symptoms, the patient’s real-time reverse transcription polymerase chain reaction (RT-PCR) test done on the 19th day returned negative results.

**Conclusions:**

Integrative therapy was found to be effective in mitigating the symptoms of COVID-19 in this patient with multiple comorbidities. Moreover, a significant improvement in blood sugar levels (not under control with modern medicine) was also achieved. Integrative therapy based on the classical texts of Ayurveda and Yoga may offer a promising and scalable treatment option for COVID-19 patients. A case series or a suitably designed randomized controlled trial is needed to assess its efficacy.

## Introduction

The coronavirus disease 2019 (COVID-19) pandemic has wreaked havoc on every aspect of human life on planet Earth. As on August 28, 2020, more than 24.4 million cases of COVID-19 have been reported in more than 227 countries and territories, resulting in more than 831,827 deaths [1]. Although there are several clinical trials underway, no cure has yet been found for COVID-19 in modern western medicine (MWM).

The presence of comorbidities such as diabetes mellitus (DM), hypertension (HTN), chronic obstructive pulmonary disease (COPD) or old age leads to poorer clinical outcomes in COVID-19 patients [2–5], and presents additional challenges in the management of the illness. Physical inactivity, high-fat diet and uncontrolled HTN in addition to superposed infection are strong risk factors for cardiovascular complications such as myocardial toxicity in COVID-19 [6, 7]. The mortality rate for hospitalized COVID-19 patients in China has been reported as 3.1% [2], while among these hospitalized patients, for those who have one or more of the nine comorbidities (DM, HTN, COPD, cardiovascular disease, cerebrovascular disease, hepatitis B infection, malignancy, chronic kidney disease [CKD], immunodeficiency), the mortality rate has been reported as 8.8% [2]. In the same cohort, the percentage of patients for whom the illness progresses to a severe stage has been reported at 16%, whereas for patients with comorbidities, the corresponding percentage is substantially higher, at 32.8%. Although the precise figures may vary with geography, the fact that the presence of comorbidities leads to poor clinical outcomes in COVID-19 patients has been independently confirmed in many studies from various countries [3, 4, 8, 9].

Ayurveda is a system of traditional Indian medicine which is based on sound therapeutic principles and has a proven history of empirical use [10, 11]. It is one of the world’s oldest holistic healing systems. According to the Ayurvedic system of medicine, a healthy person should have a stable equilibrium (congenial homeostasis) of *Doshas* (*Vata*, *Pitta*, *Kapha*—psycho-biological rhythms), *Agni* (metabolism/digestion), *Dhatu* (body tissues/elements that provide them nourishment) and *Mala* (excreta), and the well-being of senses, mind and soul [10]. Every individual has a unique combination of these constituent elements, which is known as the individual’s *Prakruti* or unique mind-body constitution (Ashtanga Hridaya, Sharira Sthana, Chapter 3, Verse 83) [12]. When an imbalance occurs in this equilibrium, it causes disease, and the Ayurvedic system of medicine seeks to remove this imbalance, to regain a healthy state [10, 11].

The occurrence of disease can be further understood as follows: the abovementioned constituent elements have a propensity to change, influenced by various factors such as the environment we live in, change in seasons, the food that is consumed, negative experiences, presence of physical toxins, irritants, microorganism or impurities, unhealthy habits and emotions. These influences distort the natural balance of these elements in an individual, increase *ama* (toxins), contributing to *vikruti* (vitiation of the constituent elements); this imbalance manifests as a lack of energy, excess mucous, inflammation, and a wide variety of dysfunction and diseases [10, 11, 13]. By using the Ayurvedic principles, the unique *Prakruti* (constitution) of the individual is assessed, the *vikruti* (vitiation) that has occurred is diagnosed and a personalized treatment plan is advised accordingly. Thus, the Ayurveda system of medicine is inherently personalized, which treats the patient by considering the individual constitution (*Prakruti*) as well as the causes of the symptoms (*vikruti*).

Classical Ayurveda texts such as *Charaka Samhita* (*Vimana Sthana*, Chapter 3) [11] and *Sushruta Samhita* (*Sutra Sthana*, Chapter 6, Verses 19, 20) [10] contain descriptions of pandemics and describe them as *Janapadoddhvansa*—Jana-pada (meaning community) + Udhvansa (meaning destruction), literally translating to “destruction of communities.” These texts offer specific guidelines with regard to the treatment of diseases that affect people during *Janapadoddhvansa*.

Yoga is yet another ancient wisdom from India, which is an extremely deep science that helps one lead a harmonious life [14]. The classical text *Yoga Vasishta* [15] (Book 6, Chapter 81) describes a healthy person as one who is free from physical disease as well as from erroneous affections of the mind. Yoga deals with the holistic principle of body-mind-soul, which proclaims that a human being can experience five dimensions of gross and subtle existence, called *Pancha kosha*, or five sheaths, namely *Annamaya* (physical body), *Pranamaya* (energy field), *Manomaya* (mental dimension), *Vijnanamaya* (related to intuitive knowledge) and *Anandamaya* (level of bliss) *kosha* (*Taittiriya Upanishad*) [16, 17]. *Prana* (the vital life force that is responsible for all the gross and subtle activities) pervades all five sheaths, and nourishes and sustains them [17]. Yogic practices are aimed at achieving the proper distribution and circulation of *Prana* within the gross and subtle realms of existence, for holistic well-being.

Ayurveda also describes the concept of *Panchaprana* or *Pancha Vayu* (five manifestations of the vital life force in the human body) (*Ashtanga Hridaya*, *Sutra Sthan*, Chapter 12, Verse 4) [12]. *Panchaprana* govern different areas of the body and different physical, neurological and mental activities. When they are functioning harmoniously, they promote the health and vitality of the body and mind, and elevate one spiritually.

Thus, both Ayurveda and Yoga are powerful sciences that are aimed at the proper modulation of *Prana* in the human being, and when administered together in an integrative manner, these can be extremely effective with regard to restoring health, as well as managing or curing diseases.

In this paper we report presumably the first high-risk case of COVID-19 (with reference to the studies available in peer-reviewed open literature), treated successfully using integrative therapy comprising Ayurveda and Yoga. Although there have been some case reports (see the section on Related Works) and theoretical works on the use of Ayurveda or Yoga for COVID-19, the authors are not aware of any published work reporting the successful treatment of high-risk cases of COVID-19 through Ayurveda or Yoga. The present case offers sufficiently supportive evidence that an integrative therapy plan based on Yoga and Ayurveda can be useful in treating even high-risk cases of COVID-19 with mild to moderate symptoms. With regard to the work ahead, a suitably designed case series or randomized controlled trial (RCT) would be advisable to further establish its efficacy for high-risk COVID-19 patients.

## Related works

There is emerging evidence that Ayurvedic treatment methodologies and herbal medicines may be effective in combating COVID-19. Girijaa and Sivan [18] describe one of the first case reports of a patient with COVID-19 treated entirely with Ayurveda. In a study using molecular dynamics simulations, Kumar *et al.* [19] report that withaferin A (Wi-A) and withanone (Wi-N), derived from a common Ayurvedic herb called *Ashwagandha*, may block the entry of the severe acute respiratory syndrome coronavirus 2 (SARS-CoV-2) into cells. Several herbs including *Tulsi* (*Ocimum sanctum*) and *Haldi* (*Curcuma longa*—turmeric) used as Ayurvedic medicines are well known for their immunomodulatory properties [20–24]. Ayurvedic treatment has also been found to be effective in other COVID-like illnesses such as influenza [25–27] and chikungunya [28]. Several studies have been conducted to understand the COVID-19 pandemic and the conditions that it causes from the perspective of Ayurveda [29–35]. Based on these observations and the study of classical Ayurveda texts, several experts have proposed the use of Ayurveda for both prophylactic [36–49] and therapeutic purposes [34, 38–41, 43, 44, 50–58] in COVID-19. Some studies have also suggested that lifestyle modifications, such as immunity-boosting food, can aid in fighting the current situation [59]. Many studies have come up with suggestions for reforms in policies enabling the adoption of Ayurveda in the treatment plan for COVID-19 patients [42, 60–62]. The Government of India, Ministry of AYUSH, has issued an advisory comprising Ayurveda-based immunity-boosting measures for self-care during the COVID-19 crisis [63], and an advisory for the general public on the use of Ayurveda (and other traditional systems of medicine) as a preventive measure for COVID-19 as well as for managing the symptoms of COVID-19 [64]. The same department also issued guidelines for Ayurveda practitioners [65] and Yoga practitioners [66] for treating COVID-19 patients. Several ongoing clinical trials are evaluating the efficacy of Ayurveda-based interventions for both prophylactic and therapeutic use in patients with mild to moderate COVID-19 symptoms.

Traditional Chinese medicine (TCM), which is based on principles similar to those of Ayurveda, has been used extensively in China for treating COVID-19. The Chinese government has actively promoted the use of TCM for management of COVID-19. As a result, several studies have been published on the use of TCM for COVID-19 [67–70]. The Chinese doctors now recommend the use of TCM along with western medicine in treating COVID-19 [71, 72]. According to Yang *et al*. [73], more than 85% of the cases in China have received TCM-based treatment. Several RCTs on TCM are presently in different stages of completion. Most of the reviews on RCTs on the efficacy of TCM for COVID-19 [74–78] report positive findings (see [79, 80] for reviews on the mechanism of action of TCM). Several countries have issued guidelines for the use of traditional medicine for COVID-19. See [81, 82] for a review of such guidelines.

Despite the above studies on TCM, caution has also been advised in the use of TCM for COVID-19. For example, Gray and Belessis [83] state that the use of TCM may cause “more harm than good.” Cyranoski [84] argues that TCM is still an unproven treatment and is being excessively promoted by certain governments. A review by Pang *et al.* [85] reported that after a comprehensive literature search, they found 26 published clinical controlled trials of TCM for COVID-19, among which 11 were RCTs with 1301 patients, but none of the RCTs were placebo-controlled or double-blinded. There was significant variation in the types of patients, interventions and outcomes in different RCTs. Also, for most of the published trials, the protocols had not been registered before case recruitment. Thus, based on the above observations, it may be concluded that TCM may offer a promising option for treating COVID-19, yet suitably designed multi-country RCTs may be needed to assess its efficacy.

In light of the above observations, the present integrative therapy plan was designed, which comprises elements of Yoga and Ayurveda, along with dietary recommendations and lifestyle modifications, as a treatment option for COVID-19. It was hypothesized that integrative therapy based on Ayurveda and Yoga would be effective in treating COVID-19 patients.

## Case presentation

### Patient information

A Delhi-based 55-year-old man (confirmed positive) COVID-19 patient (height: 5′3″; weight: approximately 70 kg) completed the online registration and consent forms on July 1, 2020, to receive Yoga and Ayurveda-based integrative therapy via telemedicine. The Ayurveda doctor and the Yoga consultant talked to the patient over the phone the same day to take his medical history and symptoms pertaining to COVID-19, and to prescribe integrative therapy-based treatment. The patient reported that his oxygen saturation value ranged between 95% and 98% (never required oxygen supplementation) and his blood pressure was under control (with medication).

#### History of the present illness

The patient was asymptomatic 3 weeks prior to the first consultation for the integrative therapy. Later, he started developing symptoms of pneumonia, after which he went to a hospital and underwent the RT-PCR test. The patient was found to be positive on June 24, 2020. Subsequently, he was prescribed the following allopathic medications for 10 days: nitazoxanide 500 mg, doxycycline 100 mg, pantoprazole 40 mg, vitamin C, multivitamin (methylcobalamin, pyridoxine and folic acid) and levocetirizine 5 mg. On July 1, 2020, at the time of the first consultation for the integrative therapy, the patient was quarantined at home (although the doctors had advised hospitalization).

#### Complaints due to present illness

##### Resolved prior to the first consultation

The patient had fever for about 10 days until June 22, 2020, after which he never had fever again. He also reported having had a dry cough and body aches earlier, both of which no longer persisted at the time of his first consultation.

##### Existing at the time of the first consultation

The patient reported experiencing occasional obstruction in the throat while speaking for a substantial duration of time. He also felt weak and sounded very low and lacking in energy. According to the patient’s description, he experienced exhaustion even in doing his own daily chores. The patient had heaviness in the abdomen (gastric upset) and often experienced a bloated feeling. Also, the patient had been having a sense of bad smell and taste, which occurred less frequently then as compared to earlier. The patient’s appetite was reduced.

#### Other existing illnesses/comorbidities

The patient was found to have the following comorbidities at the time of the first consultation. He had type 2 DM for the past 10 years and had been on allopathic medication (glimepiride 2 mg, vildagliptin 50 mg, voglibose 0.3 mg) for the same. However, despite regular use of the prescribed medicine, the illness was not very well controlled, with fasting blood sugar (FBS) mostly remaining above 200 mg/dl and postprandial blood sugar (PPBS) above 250 mg/dl, as well as renal dysfunction (as evident in his reports dated June 23, 2020). The patient was also found to be hypertensive for the past 20 years, and had been on allopathic medication (Torcilin-10); HTN was under control with the medication. He had hypothyroidism for 22 years which was being treated with allopathic medication (levothyroxine sodium 75 mcg) and was under control.

#### Family and social history

The patient had a family history of HTN in the father, grandfather and paternal uncle. There was no family history of DM. The patient had no history of smoking or taking alcohol.

Since the patient was advised through telemedicine, physical examination was not possible.

#### Occupational details

The patient is a self-employed businessman.

#### History of past illnesses

The patient had undergone cholecystectomy (removal of gall bladder) 20 years earlier.

#### Data from diagnostic tests

The data from hematology, biochemistry and ultrasound reports is as follows. The parameters marked with an asterisk (*) have values outside the reference range.

The hematology report of the patient, dated June 22, 2020, gave the following information:

- Complete blood count (white blood cell count 6.54 × $10^3$/μl; neutrophil percent 68.5%; lymphocyte percent 20.8%; monocyte percent* 9.1%; eosinophil percent* 0.8%; basophil percent 0.2%; neutrophil number 4.48 × $10^3$/μl; lymphocyte number 1.36 × $10^3$/μl; monocyte number 0.6 × $10^3$/μl; red blood cell count 5.34 × $10^3$/μl; hemoglobin concentration* 11.4g/dl; hematocrit* 34.6%; mean corpuscular volume* 64.8fL; mean corpuscular hemoglobin* 21.3pg; mean corpuscular hemoglobin concentration 32.9g/dl; red cell distribution width* 17.9%; platelet count* 50x$10^3$/μl; mean platelet volume 10.3fl; procalcitonin* 0.051%; platelet distribution width* 14%—Remarks: hypochromia, microcytosis).
- Erythrocyte sedimentation rate* 22 mm first hour.

The biochemistry report dated June 22, 2020, gave the following information:

- Glycosylated hemoglobin (HbA1c)* (high-performance liquid chromatography on D10 analyzer) 9.2%— Remarks: Average blood glucose concentration is around 210–220 mg/dl.
- Renal profile (blood urine nitrogen—kinetic ultraviolet test* 30.4 mg/dl; creatinine—kinetic color test* 1.83 mg/dl; uric acid—enzyme color test* 10.68 mg/dl; sodium* 132 mEq/l; potassium* 5.31mEq/l; calcium—color test 9.4 mg/dl; inorganic phosphorus—UV test 3.0 mg/dl—Remarks: deranged kidney function, electrolyte imbalance).

The ultrasound (whole abdomen) report, dated June 23, 2020, mentioned:

- Grade II hepatic steatosis, hepatosplenomegaly, umbilical hernia.

### Ayurvedic interpretation of the patient’s condition

#### Diagnosis

Based on the above history and discussion with the Ayurvedic doctor and Yoga consultant, the diagnosis of the patient’s illness included COVID-19, with type 2 DM, HTN, hypothyroidism and CKD. Except for the inability to perform a physical examination, no known diagnostic challenges were encountered.

#### Pathophysiology

According to Ayurvedic principles, because *jwara* (fever), *shvasa* (respiratory distress) and *kasa* (cough) are the three major symptoms of this *roga* (disease), the *roga marga* (pathway of disease) of this *roga* can be considered to be *Abhyantara* (internal origin) [18]. Because there is respiratory distress, along with other symptoms, there is *Pranavaha Sroto Dushti* (obstruction of the *Pranavaha Srotas*, that is, the subtle micro-channels in the body that are the pathways for the vital life force); also, one of the primary seats of this disease is *Uras* (chest region) [18]. Based on these observations, this disease can be characterized as *Agantuja Sannipataja Jwara*, wherein *Vata-Kapha doshas* are primarily vitiated [18].

In *Agantuja Sannipataja Jwara*, *Agantuja* implies *Agantu* (external), which is caused by *Bhoota Abhishanga* (external causative factors like a virus); this in turn causes the vitiation of all three *Doshas*, that is *Vata*, *Pitta* and *Kapha* [18]. Because of the vitiation of all three *Doshas*, the term *Sannipataja* is used [18]. As per Ayurvedic principles, the treatment of such a *Jwara* is similar to that of *Nija Jwara*, that is, one caused by vitiation of *Doshas* [18].

#### Etiology

According to Ayurvedic principles, this disease can be correlated with *Agantuja Sannipataja Jwara*, wherein *Vata-Kapha* are primarily vitiated [18]. Furthermore, this is an extremely contagious disease, caused by *Bhoota Abhishanga* (external causative factors like virus), that is characterized in Ayurvedic texts as a *Janapadoddhvansa* disease [10, 11, 18]. Classical Ayurvedic texts like *Charaka Samhita* (*Vimana Sthana*—Chapter 3) [11] and *Sushruta Samhita* (*Sutra Sthana*, Chapter 6, Verses 19, 20) [10] contain descriptions of similar pandemics, and describe them as *Janapadoddhvansa*—*Janapada*, meaning community + *Uddhvansa*, meaning destruction—literally translating to destruction of communities. The symptoms of illness during *Janapadoddhvansa*, as mentioned in classical texts, include cough, dyspnea, asthma, vomiting, nasal catarrh (common cold), headache and fever [10]. These texts also offer specific guidelines with regard to the treatment of diseases that affect people during *Janapadoddhvansa*, which include Ayurvedic medicines and spiritual practices [10, 11].

### Therapeutic intervention

This section presents the detailed treatment plan as well as the details of the subsequent compliance of the patient.

#### Treatment plan

The treatment plan comprised (1) Ayurvedic medicines, including*Giloy Ghanvati*, *Ashwagandha vati*, *Pathyadi Kwath* (*pravahi*) and *Diabecon*; (2) Yoga protocol, consisting of *Sukshma Vyayama*, breathing exercises, *Asanas*, *Shavasana*, *Pranayama* and *Dhyana*; (3) dietary modifications such as *Usha Paan*, intake of fruits and green vegetables, and avoiding cold or heavy to digest food; and (4) lifestyle modifications such as adoption of spiritual practices.

The detailed prescription of Ayurvedic medicines including the dosage, specific details about each component of the Yoga protocol, and dietary and lifestyle modifications suggested, are given below.

The following Ayurvedic medicines were prescribed: *Giloy Ghanvati* (three doses of two tablets each to be taken after meals with lukewarm water), *Ashwagandha vati* (two doses of two tablets each to be taken after meals), *Pathyadi Kwath* (*pravahi*) (15 ml mixed with an equal quantity of lukewarm water to be consumed twice a day 30 minutes after meals), and *Diabecon* (two doses of one tablet each to be taken 30 minutes before meals) (composition given in Table 1).

**Table 1:** Composition of Ayurvedic medicines

Common name	Botanical name
Giloy Ghanvati	
Giloy	*Tinospora cordifolia*
Ashwagandha vati	
Ashwagandha	*Withania somnifera*
Pathyadi Kwath (pravahi) [119]	
Harad	*Terminalia chebula*
Baheda	*Terminalia bellerica*
Amla	*Emblica officinalis*
Neem chhal	*Azadirachta indica*
Giloy	*Tinospora cordifolia*
Haldi	*Curcuma longa*
Diabecon [120]	
Shilajeet	*Asphaltum punjabianum*
Meshashringi/Gudmara/Madhunashini	*Gymnema sylvestre*
Peetashala/Vijaysar/Indian Kino Tree	*Pterocarpus marsupium*
Turmeric	
Haldi	*Curcuma longa*

The Yoga protocol designed for the patient included *Sukshma Vyayama* (subtle joint movements for upper and lower body parts), breathing exercises (four exercises with five iterations in each), *Asanas* (*Parshva Sukhasana*, *Sukhasana* twist, *Utthana Mandukasana*, *Ardha Ushtrasana*, *Meru Vakrasana*, *Ardha Halasana*—with one leg folded, *Anantasana*, *Pawan Muktasana*), *Shavasana*, *Pranayama* (sectional breathing and full yogic breathing, *Anulom-Vilom*, *Bhramari*, *Udgeet*),and *Dhyana* (mindful breathing). Online guided sessions were given for the first 14 days, after which a recorded video of the protocol (with verbal instructions and demonstration) was provided for the patient to practice independently. Looking at the anxiety level of the patient, he was also advised to practice *Yoga Nidra* [86] (guided yogic sleep and relaxation practice), for which he was provided a link to guided audio instructions (in Hindi) by Swami Satyananda Saraswati; the duration of the session was 42 minutes.

The following dietary and lifestyle modifications were also recommended to the patient: beginning the day with *Usha paan*, that is, drinking 2–3 glasses of lukewarm water, dietary intake of green vegetables, fruits, sprouts, barley, ragi flour, wheat porridge, *sahajan* (drumstick), roasted gram, *methi* (fenugreek), turmeric milk and dry fruits. Also, he was advised to refrain from cold, sour, fried, spicy or other food items that are heavy to digest; these included food items like curd, cold drinks, ice creams, chilled water, any refrigerated food items, black gram pulse (or any food item prepared with black gram), jackfruit and so on. Besides the above dietary recommendations, the patient was encouraged to undertake spiritual practices such as meditation and chanting at his convenience, for which he was provided a link to the suggested spiritual practices [87].

A detailed justification of the proposed treatment plan is provided in Additional file 1.

#### Compliance

The compliance details for the patient are given in Table 2.

**Table 2:** Compliance chart

Day	Intervention				
	Giloy	Ashwagandha	Pathyadi	Diabecon	Yoga+
	Ghanvati	Vati	Kadha		Pranayama
1	✓	✓	✓	✓	✓
2	✓	✓	✓	✓	✓
3	✓	✓	✓	✓	✓
4	✓	✓	✓	✓	✓
5	✓	✓	✓	✓	✓
6	✓	✓	✓	✓	✓
7	✓	✓	✓	✓	✓
8	✓	✓	✓	✓	✓
9	✓	✓	✓	✓	✓
10	✓	✓	✓	✓	✓
11	✓	✓	✓	✓	✓
12	✓	✓	✓	✓	✓
13	✓	✓	✓	✓	×
14	✓	✓	✓	✓	✓
15	✓	✓	✓	✓	✓
16	✓	✓	✓	✓	✓
17	✓	✓	✓	✓	×
18	✓	✓	✓	✓	✓
19	✓	✓	✓	✓	✓
20	✓	✓	✓	✓	✓
21	✓	✓	✓	✓	✓

## Results

The first consultation with the patient was on July 1, 2020. He was under home isolation during the entire course of treatment. He started observing improvement in symptoms 2 days after the beginning of the treatment. The second RT-PCR was done on July 6, and was positive. A slight modification was made in the intervention on July 9, as reported in Table 3. By the ninth day after starting the treatment, all the symptoms reported by the patient were resolved, except mild heaviness in the abdomen. The third RT-PCR done on July 14 was inconclusive, and another test was done on July 20, which was negative. Moreover, the patient’s uncontrolled blood sugar level showed significant improvement, with FBS of 90 mg/dl and PPBS of 140 mg/dl. Most importantly, the patient was in a much better state of mind (as evident in his feedback during consultations) and sounded much more confident by the end of the treatment.

**Table 3:** Progress of the patient and evolution of the treatment plan

Day	Date	FBS (mg/dl)	PPBS (mg/dl)	RBS (mg/dl)	Follow-up details
0	1/7/2020	–	–	–	Generally the blood sugar values were FBS > 200 mg/dl, PPBS > 250 mg/dl while the patient was taking allopathic medication for the same; PPBS was reported to be 230 mg/dl on 30/6/2020
1	2/7/2020	192	–	–	Symptoms reported/observed: difficulty in speech, weakness, sometimes feeling of bad smell and taste, heaviness in abdomen (due to gas formation), uneasiness in body, less appetite
2	3/7/2020	169	–	–	Improvement observed; approximately 25% relief reported in all symptoms; lightness in the body reported
3	4/7/2020	190	206	–	Doing Yoga (twice daily); feeling good, with improvement in all symptoms
4	5/7/2020	160	206	–	Approximately 50% relief reported in all symptoms; lightness in the body reported
5	6/7/2020	155	175	–	No weakness; normal appetite; taste and smell almost normal; approximately 75% relief reported. Second RT-PCR done
6	7/7/2020	140	–	110	No sore throat; no weakness; normal taste and smell; almost no other problem except mild heaviness in abdomen; approximately 80% relief reported
7	8/7/2020	140	–	110	No problem except mild heaviness in abdomen
8	9/7/2020	130	230	–	Second RT-PCR report received on 8/07/2020 was positive; slight change made in the prescription: (i) Diabecon 1 tablet in the morning and 2 tablets in the evening (previously 1 tablet in the evening); (ii) advised to take (ajwain + saunf + dhaniya) powder (1/2 spoon) with lukewarm water after meals
9	10/7/2020	110	150	–	Improvement in all problems (including heaviness in abdomen)
10	11/7/2020	110	150	–	No problem except mild heaviness in abdomen; dose of Diabecon changed to 2 tablets twice daily (patient advised to start the revised dose from 12/7/2020)
11	12/7/2020	130	140	–	No problem except mild heaviness in abdomen; started Diabecon 2 tablets twice daily
12	13/7/2020	99	112	–	No problem except mild heaviness in abdomen. Third RT-PCR done
13	14/7/2020	108	112	–	No problem except mild heaviness in abdomen
14	15/7/2020	120	165	–	No problem except mild heaviness in abdomen
15	16/7/2020	108	140	–	RT-PCR test inconclusive as per the report; no problem except mild heaviness in abdomen
16	17/7/2020	90	140	–	No problem except mild heaviness in abdomen
17	18/7/2020				No problem except mild heaviness in abdomen
18	19/7/2020				No problem except mild heaviness in abdomen
19	20/7/2020				Sample given for RT-PCR test; the patient’s allopathic doctor reduced the dosage of his diabetes medication, that is glimepiride changed from 2 mg to 1 mg; no problem except mild heaviness in abdomen
20	21/7/2020				Diabecon changed to Diabecon DS 1 tablet twice daily; no problem except mild heaviness in abdomen
21	22/7/2020				RT-PCR negative; no problem except mild heaviness in abdomen

*FBS* fasting blood sugar, *PPBS* postprandial blood sugar, *RBS* random blood sugar, *RT-PCR* real-time reverse transcription polymerase chain reaction

### Timeline of COVID-19 symptoms and treatment

A detailed description of the progress made by the patient and the medicines given during the course of the treatment is given in Table 3.

### Follow-up

Follow-up was carried out until August 01, 2020. The patient continued to report mild heaviness in the abdomen until July 26, 2020. Thereafter, beginning July 27, 2020, he reported complete relief and rejuvenation. Besides the symptoms that appeared after infection with the coronavirus, it was observed that the other ailments which the patient had a history of were also positively impacted. Table 4 summarizes the improvements observed in the reports of the patient received on August 14, 2020, as compared to those received on June 23, 2020; significant improvement can be seen in the hemoglobin A1c (HbA1c) values and renal profile parameters. In a more recent follow-up taken on October 23, 2020, the patient reported that he has continued to take the prescribed integrative therapy and is feeling complete relief of all his symptoms, including those related to his comorbidities. He further reported that, after looking at his present condition and most recent diagnostic test results, which indicated normal values for HbA1c, renal and lipid profile, his consulting physician further reduced his dosage of MWM for DM and HTN.

**Table 4:** Follow-up details

	June 23, 2020	August 14, 2020	Reference range
Blood sugar			
Glycosylated Hb (HbA1c)	9.2%	6.6%	< 6.5%
Renal profile			
Blood urea nitrogen–kinetic ultraviolet (UV) test (urease, glutamate dehydrogenase)	30.4mg/dl	11.2 mg/dl	7.94–20.09 mg/dl
Creatinine–kinetic color test	1.83 mg/dl	0.75 mg/dl	0.67–1.17 mg/dl

## Discussion

This study reports the successful treatment of a high-risk case of COVID-19 in a patient with several comorbidities, using an integrative therapy plan based on Yoga and Ayurveda, along with government-mandated modern western medicine treatment. Various unique aspects of this treatment approach are discussed below.

Since the patient’s fever was already controlled before enrollment in the integrative therapy, it is possible that he may have been in the convalescent phase at the time of enrollment. It must be noted that the Ayurveda system of medicine does not treat a disease condition in isolation; it treats a patient holistically for overall health while strengthening the body’s innate mechanisms involved in the restoration of health. The patient had uncontrolled blood sugar levels (which is known to adversely impact the immune system [88]) for the past 10 years despite MWM treatment. The rapid reduction in the blood sugar levels after the initiation of integrative therapy may have contributed to a rapid recovery from the other symptoms and provided a faster path to the patient’s overall well-being. Besides the data presented in Table 4, according to a more recent follow-up taken on October 23, 2020, the patient has continued to use the prescribed integrative therapy as an adjunct to his MWM treatment for DM and reported HbA1c within normal limits. His MWM dose for DM has been further reduced by his consulting physician.

Besides the variety and severity of physiological symptoms posed by COVID-19, it deeply affects patients psychologically as well [89, 90]. Owing to the highly contagious nature of the coronavirus, the social stigma associated with the disease and the mandated isolation period have a deep adverse impact on the already nervous patients [91]. This stress and anxiety may tend to further weaken the already compromised immune system, thereby potentially creating a vicious cycle. The patient treated in the present case was undergoing a similar situation at the time of the first consultation. With his symptoms prevailing for a long period, and two more of his family members being infected (one of them being hospitalized), the patient was in severe distress and exhibited anxiety. The integrative therapy administered to him not only helped manage his physiological symptoms, but also helped control his anxiety and stress (see for example [92, 93] for reviews on Yoga for anxiety). Within a week, an observable change in his confidence level was seen, and he appeared much more calm and peaceful. This change may be attributed to his regularity in attending the Yoga sessions, in addition to the relief experienced as his COVID-19 symptoms subsided. The authors believe that apart from boosting immunity, Yoga and *Pranayama* practices contributed immensely to his overall healing by inducing a relaxation response [94] in the body.

Promising results observed in several research studies suggest a favorable impact of Yoga on immune function, stress response, mental health and quality of life [95–100]. The usefulness of nasal irrigation, a yogic practice, in improving the symptoms and health status of patients with sinonasal disease has also been observed [101]. A key symptom associated with COVID-19 is the onset of dyspnea between the fourth and tenth days of illness [102]. Studies suggest that COVID-19 patients with hypoxia or dyspnea may have a higher risk of mortality or developing severe symptoms [103, 104]. It has been reported that a simple intervention such as prone positioning improves clinical outcomes in such cases [105, 106]. Yoga and *Pranayama* have been found helpful in cases of high-altitude hypoxia [107] and several similar interventions in pulmonary rehabilitation for patients with acute respiratory distress syndrome (ARDS)/COPD [108–114]. Owing to its role in stress reduction and immune modulation, Yoga has been proposed as a complementary therapy in the management of infectious conditions like COVID-19 [115]. Bushell *et al*. [116] suggest Yoga practices and meditation as a potential adjunctive treatment for COVID-19. In light of the above observations, and looking at the results of the present study, it is hypothesized that the integrative therapy plan, which comprises elements of Yoga and Ayurveda, along with dietary recommendations and lifestyle modifications, would be effective in treating COVID-19 patients, either as an adjunct to MWM or as a stand-alone treatment. A case series involving a larger number of high-risk patients or a suitably designed RCT is needed to systematically assess its efficacy.

## Conclusion

The success of the integrative therapy in this high-risk case of COVID-19 is evident in that the patient not only recovered from the symptoms, but a sense of overall well-being was bestowed on him.

This successfully treated case certainly calls for more well-designed studies on the proposed integrative therapy approach, possibly leading to its adoption in the standard treatment provided to COVID-19 patients.

## Patient’s perspective on the treatment

After recovery from symptoms and a negative RT-PCR report, a subjective feedback was taken from the patient to understand his perspective on the intervention. The patient expressed immense satisfaction and a sense of confidence. He was amazed and delighted to find that besides aiding in a speedy recovery from the symptoms of COVID-19, including weakness, anxiety and depression, Yoga and Ayurveda had managed to control his blood sugar level as well. The normal sugar levels observed by the end of the treatment were astounding given that regular allopathic medication could not achieve this. The patient was so convinced about the efficacy of Yoga and Ayurveda that he suggested his spouse (who was also tested positive for COVID-19 and had been pursuing only allopathic medication) seek Ayurvedic consultation. Most importantly, he is determined to adopt Yoga in his lifestyle.

## Supplementary Information

**Additional file 1:** Justification for the prescribed treatment.

## Data Availability

All necessary data are available (in de-identified form) from the corresponding author on reasonable request.

## Abbreviations

SARS-CoV-2: Severe acute respiratory syndrome coronavirus 2
DM: Diabetes mellitus
HTN: Hypertension
CKD: Chronic kidney disease
FBS: Fasting blood sugar
PPBS: Postprandial blood sugar
RT-PCR: Real-time reverse transcription polymerase chain reaction
COPD: Chronic obstructive pulmonary disease
ARDS: Acute respiratory distress syndrome
TCM: Traditional Chinese medicine
RCT: Randomized controlled trial
QRT: Quick relaxation technique
DRT: Deep relaxation technique
