# Supplementary material for: The use of integrative therapy based on Yoga and Ayurveda in the treatment of a high-risk case of COVID-19/SARS-CoV-2 with multiple comorbidities: a case report
Source: J Med Case Rep. 2021 Feb 24;15:95. doi: 10.1186/s13256-020-02624-1 (PMC7903378; doi:10.1186/s13256-020-02624-1)
Supplement: Supplementary file 1 — Additional file 1: Justification for the prescribed treatment. [file 13256_2020_2624_MOESM1_ESM.pdf]

Additional file 1

January 28, 2021

Justification for Ayurvedic Medicines

Table 1 lists the detailed composition of the prescribed Ayurvedic medicines along with the botanical names of their ingredients. We next provide the justification for prescribing these medicines, based on the classical Ayurveda texts.

Giloy is known to have the following properties [1]: *Tridosha shamaka* (pacifies all the three *doshas*, i.e. *Vata*, *Pitta*, *Kapha*), *Deepan Pachan* (*Rasa Dhatu*, after proper metabolism, converts to the next *Dhatu*, i.e. *Rakta* or blood; undigested part of *Rasa Dhatu* acts as toxin, which is called *Ama Dosha*; this toxin is responsible for the blockage of micro-channels, i.e. *srotas*, leading to diseases; the *Deepan Pachan* property provides for enhancement of digestive fire for digestion of the *Ama Dosha*, thereby freeing the body of toxins), *Jwaraghna* (antipyretic), *Pramehahara* (anti-diabetic), *Hrid-daurbalya nashak* (useful in the treatment of heart problems), *Yakrit vikar nashak* (useful in the treatment of liver related problems), *Kasa roga nashak* (useful in the treatment of cough related problems), *Raktashodhaka* (blood purifier), and *Rasayan* (rejuvenating and removes weakness). Thus, these properties of *Giloy* (which is the sole ingredient of *Giloy Ghanvati*) justify its use in the management of various symptoms of COVID-19.

Ashwagandha has the following properties [1]: *Kapha*, *Vata shamak* (pacifies *Kapha* and *Vata doshas*), *Balya* (provides strength), *Vringhan* (nourishing effect), *Rasayan* (rejuvenating and removes weakness), *Deepan* (appetizer; and enhancer of the digestive fire), *Anuloman* (carminative, i.e., removes blockage in the gastro-intenstinal tract resulting in easy passage of *Vata* such as flatus), *Shwasahara* (helpful in cases of breathlessness or dyspnea), and *mootral* (dieuretic, i.e., helpful in kidney related problems). These properties justify the use of *Ashwagandha vati* for the presented case.

The properties of the ingredients of *Pathyadi Kwath* are as follows [2].

- *Harad* has the properties of [1]: *Tridosha shamaka* (pacifier of all the three *doshas*, i.e. *Vata*, *Pitta*, *Kapha*; especially useful in *Vata* disorders), *Balya* (strength providing), *Medhya* (nervine tonic), *Deepan Pachan* (enhances the digestive fire for digestion of the *Ama Dosha*, as described above), *Yakrit uttejak* (increases liver efficiency), *Jwaraghna* (antipyretic), *Mootral* (dieuretic, i.e., useful in kidney related problems), *Anuloman* (carminative, i.e., removes blockage in the gastro-intenstinal tract resulting in easy passage of *Vata* such as flatus) and *Mridu-rechan* (mild purgative).

- *Baheda* possesses the following properties [1]: *Tridoshahara* (pacifies all the three *doshas*, i.e. *Vata*, *Pitta*, *Kapha*, and is especially useful in *Kapha* disorders), *Jwaraghna* (antipyretic), *Kasa roga nashak* (treats cough related problems), *Shwas roga nashak* (helps in breathlessness, dyspnea), *Swarabheda nashak* (helps eliminate hoarseness of voice), *Pratishyaya nashak* (useful in common cold), *Dhatuvardhak*, (nourishes body tissues, especially *Rasa* - lymph, plasma, etc., *Rakta* or blood, *Mansa* or muscles, *Meda* or fat and *Dhatus*), *Deepan* (appetizer; and enhancer of the digestive fire), and *Anuloman* (carminative, i.e., removes blockage in the gastro-intenstinal tract resulting in easy passage of *Vata* such as flatus).
- *Amla* has the following properties [1]: *Tridoshahara* (pacifies all the three *doshas*, i.e. *Vata*, *Pitta*, *Kapha*; especially useful in *Kapha* disorders), *Deepan* (appetizer; and enhancer of the digestive fire), *Anuloman* (carminative, i.e., removes blockage in the gastro-intenstinal tract resulting in easy passage of *Vata* such as flatus), *Rochan* (removes anorexia, i.e., unwillingness to eat and reduced appetite), *Rasayan* (removes weakness and is rejuvenating), *Balya* (strength providing), *Medhya* (nervine tonic), *Mootral* (dieuretic), *Jwaraghna* (antipyretic), *Pramehaghna* (used to treat *Madhumeha* or diabetes), *Yakrit uttejak* (enhances liver efficiency), *Kasa roga nashak* (treats cough related problems), *Shwas roga nashak* (useful in breathlessness, dyspnea) and *Hridroga nashak* (used to treat heart problems).
- *Neem chhal* is known to have the following properties [1]: *Kapha*, *Pitta shamak* (pacifies *Kapha* and *Pitta Doshas*), *Rochan* (removes anorexia, i.e., unwillingness to eat or reduced appetite), *Yakrit uttejak* (enhances liver efficiency), *Rakta shodhak* (blood purifier), *Prameha nashak* (used to treat *Madhumeha*, i.e., diabetes), *Ama pachak* (digests the *Ama Dosha*, i.e. the toxins in the body, as described above under *Giloy*), *Jwaraghna* (antipyretic), *Kasa roga nashak* (used to treat cough related problems), and *Dhatukshaya* (prevents depletion of *Dhatus*).
- The properties of *Haldi* are as follows: *Kapha*, *Vata shamak* (pacifies *Kapha* and *Vata Doshas*), *Pitta rechak* (removes vitiated *Pitta Dosha*), extremely useful for *Prameha* (useful in disorders like diabetes), *Ama pachak* (digests the *Ama Dosha*, i.e. the toxins in the body, as described above under *Giloy*), *Jwaraghna* (antipyretic), *Kasa roga nashak* (used to treat cough related problems), *Shwas roga nashak* (useful in breathlessness, dyspnea), *Anuloman* (carminative, i.e., removes blockage in the gastro-intenstinal tract, resulting in easy passage of *Vata* such as flatus), and *Rochak* (removes anorexia, i.e., unwillingness to eat or reduced appetite).
- The properties of *Giloy*, another ingredient of *Pathyadi Kwath*, have been mentioned above under *Giloy Ghanvati*.

Thus, the above ingredients of *Pathyadi Kwath* were expected to help the patient with the gastric issues he had been experiencing, resolve the bad taste and loss of appetite, enhance his digestion and rejuvenate him, remove weakness, as well as help in other symptoms of the disease.

The ingredients of the tablet *Diabecon* [3] have been discussed below. This was prescribed to address the not-well-controlled blood glucose levels of the patient.

- *Shilajeet* is known to possess the following properties [3, 4]: *Vata*, *Pitta hara* (pacifies *Vata* and *Pitta* Doshas), *Rasayan* (rejuvenates and removes weakness), *Ama pachak* (digests the *Ama Dosha*, i.e. the toxins in the body, as described above under Giloy), *Kapha rog nashak* (useful in *Kapha* related disorders), *Mootra roga nashak* (useful in urine related problems), useful in *Prameha* (useful in disorders like diabetes), *Kasa roga nashak* (used to treat cough related problems), and *Shwas roga nashak* (useful in breathlessness, dyspnea). It is also known to reduce hepatic glucose production, prevent hyperglycemia, protect b-cells of pancreas, and promote unrestricted endogenous insulin action [3].
- *Meshashringi/Gudmara/Madhunashini* has the following properties [3, 4]: *Kapha*, *Vata shamak* (pacifies *Kapha* and *Vata Doshas*), especially useful in *Madhumeha* (diabetes) - works by increasing the secretion of insulin and reducing the blood sugar level, *Mootral* (dieuretic, useful in kidney related problems), *Madhurya nashak* (removes the sweet taste of the tongue), *Deepan* (appetizer, enhances the digestive fire), *Yakrit uttejak* (enhances liver efficiency), *Pratishyaya nashak* (useful in common cold), *Kasa roga nashak* (used to treat cough related problems), and *Shwas roga nashak* (useful in breathlessness, dyspnea). The main constituent of *Meshashringi* is gymnemic acid that has anti-diabetic properties and reduces excess blood sugar; it also has a regenerative effect on the beta cells of pancreas and is insulinotropic, i.e. stimulates insulin's production and activity; it temporarily decreases sugar craving by removing the taste for sugar and enhances the activity of enzymes that are responsible for the absorption and utilization of glucose [3].
- *Peetashala/Vijaysar/Indian Kino Tree* has the properties of [3, 4]: *Kapha*, *Pitta shamak* (pacifies *Kapha* and *Pitta Doshas*), *Madhumeha hara* (useful in diabetes), *Rasayan* (rejuvenates and removes weakness), *Rakta shodhak* (blood purifier). It has the property of inhibiting alpha-glucosidase; it regularizes primary metabolic enzymes that are involved in carbohydrate metabolism [3].

The patient was also advised to consume turmeric milk, given the beneficial properties of *haldi* (given above).

Justification for Yoga Protocol

The *Yoga* component of the Integrative Therapy was designed to achieve maximum benefits considering the co-morbidities, COVID-19 symptoms and anxiety. The protocol began with *Sukshma Vyayama* (micro/subtle joints movements) that are preparatory practices to relieve joint stiffness and muscle tension, and also bring about relaxation. This was followed by *breathing techniques*, which improve the lung function, address weakness and reduce anxiety. Next in the protocol were *Yogasanas* (*yogic* postures) selectively picked to improve lung capacity and flexibility, enhance blood circulation, and bring about a deep sense of calmness. Postures involving intense backward bend were deliberately avoided since the patient has umbilical hernia. Following the *asanas* were *relaxation techniques* that counter stress response in the body. Stress raises blood glucose levels, suppresses the immune system, and increases blood pressure; relaxation techniques, such as Quick Relaxation Technique (QRT) [5], Deep Relaxation Technique (DRT) [5] and *Yoga Nidra* [6] are some powerful tools that reduce stress and anxiety. Given the anxiety and stress that the

patient was undergoing, relaxation techniques were expected to bring about overall healing. The *yoga* sessions always concluded with *Pranayama* and Mindfulness meditation, which are known to reduce anxiety and improve lung function.

Author details

References

1. Sharma, P.V.: Dravyaguna Vijnana, Volume II (vegetable Drugs) (Hindi). Chaukhambha Bharati Academy, Varanasi, Uttar Pradesh, India (2001)
2. Sandu Pharmaceuticals Ltd.: Pathyadi Kadha. Retrieved August 8, 2020 from http://www.sandu.in/pathyadi.html
3. Himalaya: Diabecon. Retrieved August 8, 2020 from https://himalayawellness.in/products/diabecon
4. Jha, C.B.: Ayurvediya Rasashastra, p. 325. Chaukhambha Surabharati Prakashan, Varanasi, India (2000)
5. Dr. H R Nagendra, D.R.N.: New Perspectives In Stress Management. Swami Vivekananda Yoga Prakashana, Bangalore, India (June 1986)
6. Saraswati, S.S.: Yoga Nidra. Yoga Publications Trust, Munger, Bihar, India (2015)
